# *ENG* mutational mosaicism in a family with hereditary hemorrhagic telangiectasia

**DOI:** 10.1002/mgg3.361

**Published:** 2017-12-14

**Authors:** Pernille M. Tørring, Anette D. Kjeldsen, Lilian Bomme Ousager, Klaus Brusgaard

**Affiliations:** ^1^ Department of Clinical Genetics Odense University Hospital Odense Denmark; ^2^ Department of Otorhinolaryngology Odense University Hospital Odense Denmark

**Keywords:** ENG, genetic testing, hereditary hemorrhagic telangiectasia, HHT, mosaic, mosaicism, mutational mosaicism

## Abstract

**Background:**

Hereditary hemorrhagic telangiectasia (HHT) is an autosomal dominant genetic disorder caused by mutations in *ENG*,*ACVRL1,* or *SMAD4*. Around 90% of HHT patients present with a heterozygous pathogenic genetic variation. Almost all cases of HHT have a family history. Very few cases are de novo or mosaicism. We describe a case with mutational mosaicism that would not be observed in the clinical routine when using Sanger sequencing or a NGS read coverage below app. 100.

**Methods:**

DNA was extracted from peripheral blood leukocytes, and buccal swabs. The coding region, exon–intron boundaries, and the flanking sequences of the genes were sequenced by NGS.

**Results:**

The proband had clinical HHT fulfilling the Curaçao criteria and genetic testing identified a frameshift mutation in *ENG*. The mother of the proband, also with clinical HHT, was found negative when analyzing DNA from blood for the familial mutation using Sanger sequencing. Analyzing her DNA by NGS HHT panel sequencing when extracted from both peripheral blood leukocytes, and cheek swabs, identified the familial *ENG* mutation at low levels.

**Conclusion:**

We provide evidence of *ENG* mutational mosaicism in an individual presenting with clinical HHT. These findings illustrate the importance of considering mutational mosaicism.

## 1 INTRODUCTION

Hereditary hemorrhagic telangiectasia (HHT), also known as Osler–Weber–Rendu disease, is an autosomal dominant hereditary disorder characterized by a variety of clinical manifestations due to the presence of multiple mucocutaneous telangiectases and arteriovenous malformations (AVMs) in internal organs, most commonly lungs, liver, and cerebrum. The most frequent clinical manifestation is spontaneous and recurrent epistaxis, affecting more than 95% of all HHT patients. Pulmonary arteriovenous malformations (PAVMs) are observed in about one‐third of all HHT patients (Kjeldsen, Oxhøj, Andersen, Green, & Vase, 2000), and can cause cerebral abscess or stroke due to paradoxical embolism (Kjeldsen et al., 2000; Mathis et al., 2012). Cerebral AVMs are present in at least 10% of HHT patients and hepatic AVMs are common, but rarely symptomatic (Dupuis‐Girod, Bailly, & Plauchu, 2010; Faughnan et al., 2011).

HHT is a genetically heterogeneous disorder caused by mutations in three known genes: *ENG* (OMIM 131195), *ACVRL1* (OMIM 601284), and *SMAD4* (OMIM 600993).

HHT is a clinical diagnosis, according to the Curaçao criteria (Shovlin et al., 2000). In approximately 85% of the HHT patients a mutation in either *ENG* or *ACVRL1* (Torring, Brusgaard, Ousager, Andersen, & Kjeldsen, 2013) can be identified at mutation analysis. Only around 2% of HHT patients have mutations in *SMAD4* (Gallione et al., 2004, 2006). In NGS analysis of these genes, usually patients present with a pathogenic genetic variant in a heterozygous state with an equal representation of the two alleles.

Mosaicism is defined as the presence of two or more cell lines with different genotypes in one individual, developed from a singular fertilized egg. Levels of mosaicism across tissues and body locations can show surprising variability, even within the same embryonic lineage. This variation likely results from interplay between mutation timing, cell migration and determination during development, and tissue‐specific cell‐autonomous selective effects (Campbell, Shaw, Stankiewicz, & Lupski, 2015). Individuals with mutational mosaicism may have none, reduced or full disease severity. Mutational mosaicism in HHT has been described although it is very rare. A review of the literature (August 2017) reveals four papers describing mosaicism in HHT, confirmed or suspected by Sanger sequencing (Best et al., 2011; Eyries et al., 2012; Lee et al., 2011; McDonald et al., 2011). Mutational mosaicism confirmed by NGS, which is much more precise, has, to our knowledge, only in very few cases been described in the HHT genes.

Here we show the presence of an *ENG* mutational mosaicism that would not be observed in the clinical routine if using Sanger sequencing or a NGS read coverage below app. 100.

## 2 MATERIALS AND METHODS

Danish HHT patients are referred to the Danish HHT Center for clinical and genetic evaluation as well as for treatment for HHT manifestations, especially embolization of PAVM. This was also the case for the patients included in this manuscript.

### 2.1 Ethical compliance

Both the proband and her mother have agreed to publication of photographs and clinical information.

### 2.2 DNA analysis

The *ENG* gene (RefSeq: NM_001114753.1) was sequenced with targeted NGS HHT panel using the Agilent targeted sequence capture method followed by sequencing on the Illumina HiSeq1500 NGS platform (Illumina Inc,San Diego, CA). The sequencing design of the assay utilized oligonucleotides that were designed using Agilent SureDesign to capture all exons, 5′‐ and 3′‐UTR, and 50 kb into the 5′‐ and 3′‐ UTR, and 50 kb into the flanking regions of the exons. A SureSelectXT Reagent kit (Agilent Technologies Denmark ApS, Glostrup, Denmark) was used and run on a single lane using paired‐end sequencing at 2 × 100 bp.

Our usual algorithm used in the HHT diagnostics laboratory is NGS of an HHT panel (*ENG, ACVRL1* and *SMAD4*), findings are confirmed by Sanger sequencing.

## 3 RESULTS

### 3.1 Patients

The proband is a female patient, 38 years of age: Nosebleeds began at the age of three, but have never occurred more than a couple of times a month. At the age of 10 a left‐sided pneumonectomy was performed due to severe desaturation caused by several large pulmonary arteriovenous malformations. The teenage years were generally uneventful, although some dyspnea was still present. With age, the dyspnea got worse and the patient experienced coughing. At time of referral to the Danish HHT Center (age 38) clinical examination revealed a few telangiectatic lesions, which were identified at the fingertips and the lips. Further a small cerebral AVM in the right occipital lobe, too small for treatment, was diagnosed.

**Figure 1:**
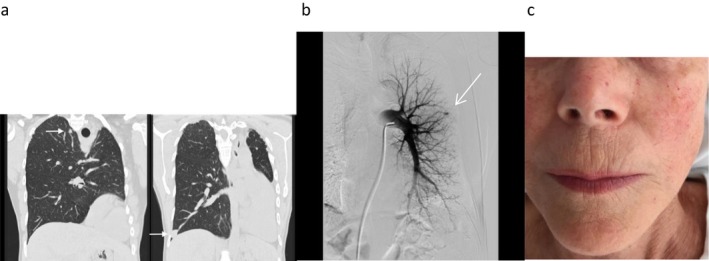
(a) CT scan of the lung of the proband. Two residual pulmonary AVMs (arrows), and only one right lung (left‐sided pneumonectomy due to severe pulmonary AVM at age 10). (b) Pulmonary angiography of the mother, with one large PAVM just prior to embolization. (c) The face of the mother at age 76, with few but typical telangiectatic lesions

Screening for pulmonary arteriovenous malformations was performed and 4 pulmonary AVMs at treatable size was diagnosed along with several small ones, too small for treatment, in the right lung (age 38). Embolization was performed at three occasions, and after the last embolization, which were at our HHT Center, saturation increased to 98% (Figure 1a: CT before last embolization). The patient is still in follow‐up.

The mother of the proband was 76 years old when she was referred to the HHT center. At the age of 18 thoracic surgery with removal of a pulmonary AVM had been performed. She never attended any follow‐up visits. She was generally in good health without dyspnea, but at the age of 74 she experienced a brain abscess, which was treated surgically. She recovered almost fully, but some deficit such as memory loss persisted. At the same time oxygen saturation was 93% and a single large PAVM was diagnosed and embolized (Figure 1b), which resulted in an oxygen saturation increase to 99%. After embolization, contrast echocardiography showed full occlusion of the PAVM. She suffered from nosebleeds once or twice a week, but treatment has not been necessary. She did not have anemia and there have been no signs of cerebral AVM at cerebral CT scan. Clinical examination, at age 76, revealed few, but typical telangiectatic lesions (Figure 1c).

Both HHT patients fulfill the Curaçao criteria (4 of 4 clinical criteria).

The other family members: The sister of the proband had no symptoms of HHT and did not carry the *ENG* mutation. The parents and siblings of the probands’ mother had no history of HHT‐related symptoms.

### 3.2 Molecular analysis

The proband was submitted to the HHT Center in Odense. She fulfilled the Curaçao criteria and genetic germline testing on a blood sample, was performed. A heterozygous genetic variant, c.704dupC; p.Val236Glyfs*98, was identified in the *ENG* gene (read count 182/333 (55%)) and confirmed by Sanger sequencing. No other mutation was identified in our NGS HHT panel encompassing the genes *ENG, ACVRL1,* and *SMAD4*. The variant c.704dupC was not previously described in the HHT Mutations Database (http://arup.utah.edu/database/ENG/ENG_display.php) or HGMD (Human Gene Mutation Database). However, as it is a truncating frameshift mutation, it was believed to be pathogenic.

**Figure 2:**
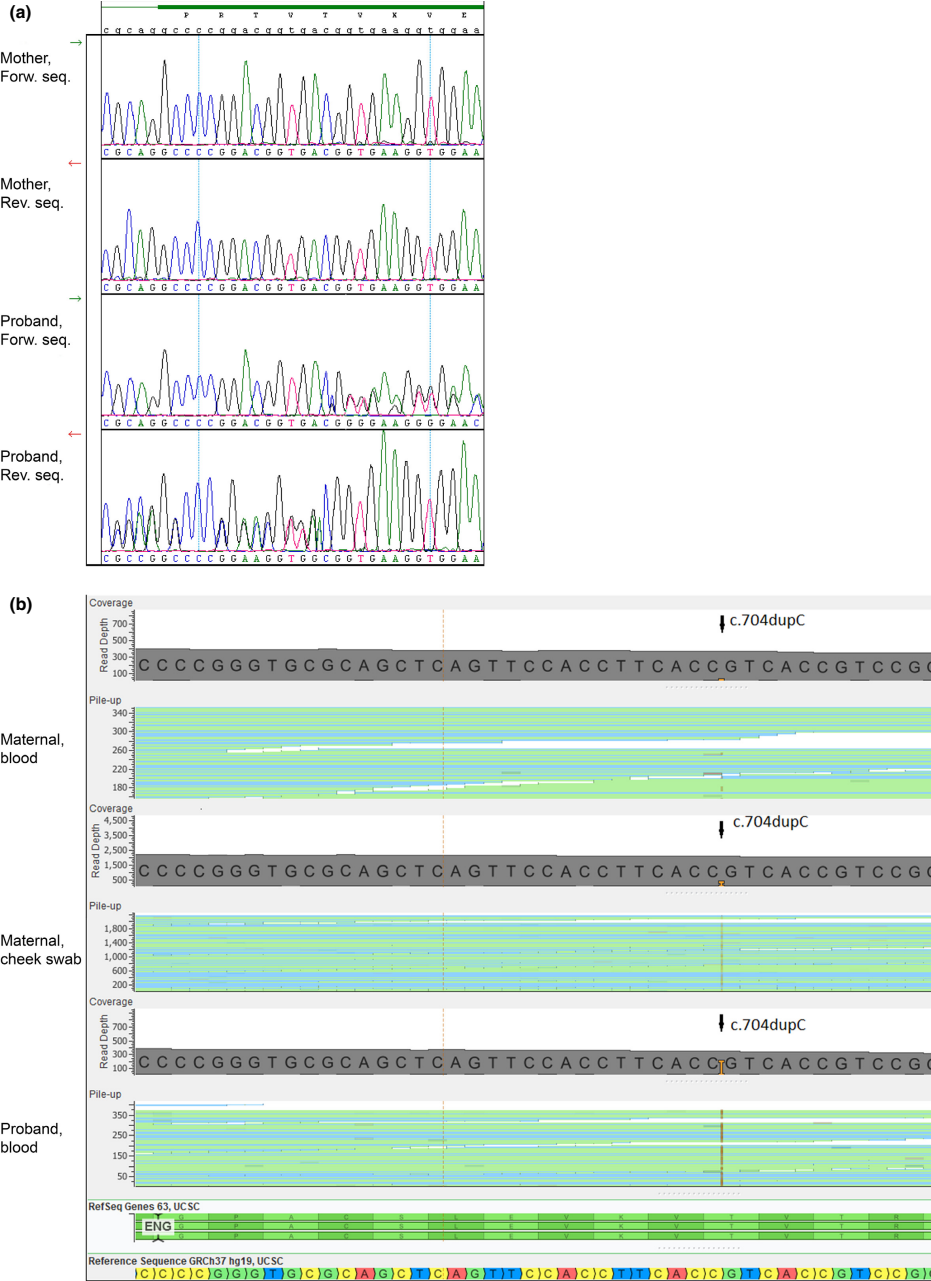
(a) Sanger sequence. Lower two rows forward and reverse sequence from the proband. Upper two lanes sequence of DNA extracted from maternal blood. (b) NGS panel. Lower row sequence from the proband. Upper two lanes sequence of DNA extracted from maternal blood (top) and cheek swab (middle)

Her mother, also presenting with clinical HHT, was found negative when analyzing for the familial mutation using Sanger sequencing. Suspecting mosaicism, we analyzed her DNA, by NGS *ENG* sequencing, extracted from both peripheral blood leukocytes, and cheek swabs. In the blood c.704dupC was identified in 7 of 364 reads, providing evidence for mutational mosaicism with the percentage of the variant allele at 2%. In DNA from cheek swabs c.704dupC was identified in 257 of 2,285 reads representing mutational mosaicism of 11% (Figure 2 and Table 1). Sanger sequencing of both tissues showed normal wildtype and did not reveal mosaicism.

**Table 1:** Presentation of allelic read counts of blood sample from the proband and blood and cheek swab samples from the mother, respectively

	Proband	Mother	
	Blood	Blood	Cheek swab
Variant	182	7	257
Reference (wildtype)	151	357	2,028
Mosaic percentage	55%	2%	11%

The family history was consistent with the mutation being de novo in the mother.

## 4 DISCUSSION

We here present NGS sequencing data showing mosaicism for a pathogenic genetic variation in an individual presenting with symptoms of HHT. The daughter in this case had very severe affection of her lungs. The mother also had clear symptoms of HHT, but less severe than the index patient. She had less severe PAVM of the lungs, though she was unfortunate to have a cerebral abscess due to her PAVM. Both patients fulfilled the Curaçao criteria (both had 4 of 4 clinical criteria), which, for the mother, was a bit surprising noting the low levels of mutational mosaicism. This case illustrates the difficulty of counseling mosaic individuals as the severity of symptoms cannot be predicted from the level of mosaicism, especially as we cannot evaluate all tissues. As the daughter inherited the mutation the mosaicism was also present in the mother's germinal tissue, and, thus, we must presume that the mutation occurred rather early on in the embryogenesis of the mother. Furthermore, the level of mosaicism in the germinal tissue cannot be predicted from the level of mosaicism in blood or other tissues, which makes it difficult to assess the risk of transmitting the mutant allele to offspring.

This finding illustrates the importance of considering investigation for mosaicism in HHT cases, with parents that have no sign of HHT, and who is found negative for pathogenic genetic variants in either of the candidate genes. The low levels of mosaicism found in the presented case could not be detected by Sanger sequencing in the two different tissues. In our analysis NGS sequencing allowed convincing detection of mutational mosaicism at the 2% level. The mosaicism was identified after the familial variant had already been identified in the daughter, which enhanced the probability of detection.

Even though mosaicism and de novo mutations are rare in HHT, this study exemplifies the importance of considering mosaicism, also in patients or families with HHT.

## CONFLICT OF INTEREST

The authors declare that they do not have any conflicts of interests.
